# Health care utilization among complementary and alternative medicine users in a large military cohort

**DOI:** 10.1186/1472-6882-11-27

**Published:** 2011-04-11

**Authors:** Martin R White, Isabel G Jacobson, Besa Smith, Timothy S Wells, Gary D Gackstetter, Edward J Boyko, Tyler C Smith

**Affiliations:** 1 Department of Defense Center for Deployment Health Research at the Naval Health Research Center, San Diego, CA, USA; 2 Analytic Services, Inc. (ANSER), Arlington, VA, USA; 3 Seattle Epidemiologic Research and Information Center, Veterans Affairs Puget Sound Health Care System, Seattle, WA, USA

## Abstract

**Background:**

Complementary and Alternative Medicine use and how it impacts health care utilization in the United States Military is not well documented. Using data from the Millennium Cohort Study we describe the characteristics of CAM users in a large military population and document their health care needs over a 12-month period. The aim of this study was to determine if CAM users are requiring more physician-based medical services than users of conventional medicine.

**Methods:**

Inpatient and outpatient medical services were documented over a 12-month period for 44,287 participants from the Millennium Cohort Study. Equal access to medical services was available to anyone needing medical care during this study period. The number and types of medical visits were compared between CAM and non-CAM users. Chi square test and multivariable logistic regression was applied for the analysis.

**Results:**

Of the 44,287 participants, 39% reported using at least one CAM therapy, and 61% reported not using any CAM therapies. Those individuals reporting CAM use accounted for 45.1% of outpatient care and 44.8% of inpatient care. Individuals reporting one or more health conditions were 15% more likely to report CAM use than non-CAM users and 19% more likely to report CAM use if reporting one or more health symptoms compared to non-CAM users. The unadjusted odds ratio for hospitalizations in CAM users compared to non-CAM users was 1.29 (95% CI: 1.16-1.43). The mean number of days receiving outpatient care for CAM users was 7.0 days and 5.9 days for non-CAM users (*p *< 0.001).

**Conclusions:**

Our study found those who report CAM use were requiring more physician-based medical services than users of conventional medicine. This appears to be primarily the result of an increase in the number of health conditions and symptoms reported by CAM users.

## Background

Complementary and alternative medicine (CAM) is a term used to describe a wide variety of procedures, substances, and approaches for treating symptoms, illnesses, and injuries, as well as promoting good health. CAM therapies include a broad spectrum of ancient to new-age approaches that purport to prevent and/or treat numerous symptoms and medical conditions. Typically they are not considered part of conventional medicine, nor are they usually taught at U.S. medical schools [1]. In 2007, approximately 4 out of 10 adults in the United States reported using some form of CAM therapy in the past 12 months [2]. Similarly, in the United Kingdom and Australia, 46%-48% of adults reported using one or more CAM therapies in their lifetime [3,4]. The fact that CAM is becoming more widely accepted in the United States and abroad has inspired a body of literature directed at examining who uses CAM and for what reasons [5,6].

A number of studies have also looked at CAM in U.S. military populations and found it to be fairly consistent with that of civilians. Typical reasons cited for choosing CAM among military populations include current high daily stress, impact of military life on physical or mental health, physician-diagnosed chronic illnesses, and the potential side effects from prescription medications. Motivation for CAM use may also involve the realization that conventional care may not adequately address chronic conditions, which are often reported by those using CAM [7]. Although there is no shortage of studies involving CAM use among various defined populations in the literature, very few have considered health care utilization patterns among CAM users compared with nonusers. A recent study reported more frequent outpatient visits to physicians among those who used CAM compared to those who did not, but no difference was noted in the rate of hospitalization [8]. Additionally, Gray et al. found that CAM users reported more physical and emotional limitations, pain, and dysthymia, but were no more likely to have reported a chronic condition than nonusers [9]. A number of studies have also shown that CAM users tend to be individuals who have more comorbid, non-life-threatening health problems than nonusers [10-14]. In a cohort of military personnel, those who reported CAM use also reported a greater number of comorbidities and poorer overall health than those not reporting CAM use [15]. Findings such as these suggest those who choose CAM therapies may also have greater use of both unconventional and conventional medical services [16,17]. Understanding the health care utilization patterns of those who use CAM in a large active-duty military cohort could also increase our understanding of the health care requirements of this particular population of patients and help quantify their overall consumption of medical services.

Additionally, our study has the advantage of capturing both inpatient and outpatient care in a large population of participants who have equal access to high-quality medical services.

## Methods

Prior to the start of the conflicts in Afghanistan and Iraq, the Department of Deployment Health Research at the Naval Health Research Center launched the Millennium Cohort Study to assess any potential long-term health effects of military service. The survey questionnaire consists of approximately 450 questions concerning the health and well-being of the cohort participants and has been described elsewhere in detail [18,19]. Also incorporated into the baseline and subsequent surveys is the Medical Outcomes Study Short Form 36-Item Health Survey for Veterans (SF-36V) [20], a modified version of the Medical Outcomes Study 36-Item Short Form Health Survey (SF-36). This 36-Item questionnaire measures health functioning on eight scales, and is among the most widely used measure of quality of life [21].

### Study Design

Participants were randomly selected from all U.S. military personnel on rosters as of October 2000. Reserve and National Guard members, those previously deployed and women were oversampled to ensure sufficient power to detect differences in these smaller subgroups. Beginning in 2004, survey questions regarding CAM use were expanded to include 12 specific measures of CAM (described below in greater detail). Only those service members on active duty (44,287) were included in this study, since Reserve and National Guard personnel are only eligible for military health care when on active status and some may have been inactive during our study period. Consequently their inpatient and outpatient care would not have been captured through our review of military records while inactivated.

This research has been conducted in compliance with all applicable federal regulations governing the protection of human subjects in research and was approved by the Institutional Review Board of the Naval Health Research Center (protocol NHRC.2000.0007).

### Data Sources

In addition to our longitudinal survey instrument, other data sources include the Standard Inpatient Data Record (SIDR), which is an electronic database of standardized discharge information for any hospitalizations within the military health care system. These data contain a summary of discharge information, including date of admission and discharge, up to eight procedural codes, and up to eight individual discharge diagnoses for each hospitalization. Specific diagnoses are coded according to the *International Classification of Diseases, 9th Revision, Clinical Modification *(ICD-9-CM) [22]. Hospitalizations that occur outside of the Department of Defense (DoD) military health care system are available through the DoD TRICARE Management Activity's Health Care Service Record, and were used to ascertain DoD-reimbursed hospitalizations of active-duty personnel. Hospitalizations for complications of pregnancy, childbirth, and the puerperium were excluded from analyses examining overall odds of hospitalization in relation to CAM use but included in analyses describing odds of hospitalization for specific diagnoses by CAM use.

For ambulatory data, we used the Standard Ambulatory Data Record to capture outpatient visits. These data are generated by military treatment facilities and include for each outpatient visit up to four diagnoses using ICD-9-CM codes. Electronic military personnel files maintained by the Defense Manpower Data Center were also used to ascertain demographic information, including date of birth, marital status, sex, race/ethnicity, occupation, service branch, service component, education level, and pay grade. Self-reported survey data were used to ascertain body mass index (BMI = weight in kilograms/height in meters squared), smoking status, alcohol consumption, and Mental and Physical Component Summary scores from the SF-36V [23]. Instances of both inpatient and outpatient care were captured for the 12-month period following each subject's enrollment into the study.

### CAM Assessment

We used 12 specific questions from the survey to assess CAM use. While these questions do not encompass the full spectrum of CAM use, they include those items believed to provide a clearer distinction between CAM and conventional medicine [1,5,10,24]. Our survey asked, "Other than conventional medicine, what other health treatments have you used in the last 12 months?", with the following options available as yes/no responses: acupuncture, biofeedback, chiropractic care, energy healing, folk remedies, herbal therapy, high-dose megavitamin therapy, homeopathic remedies, hypnosis, massage therapy, relaxation, and spiritual healing. For the purposes of these analyses, acupuncture, biofeedback, chiropractic care, energy healing, folk medicine, hypnosis, and massage therapy were grouped together as practitioner-assisted CAM therapies; herbal therapy, high-dose megavitamin therapy, homeopathic remedies, relaxation, and spiritual healing were grouped together as self-administered CAM therapies.

### Statistical Analysis

Chi-square tests were used to examine demographic and military characteristics in relation to practitioner-assisted, self-administered or no CAM use, with *p *< 0.05 considered statistically significant. Hospitalization rate was calculated as number of first hospitalizations divided by total number of subjects and expressed as the annual number of first hospitalizations per 1,000 persons in relation to CAM use. Multivariable logistic regression was used to compare unadjusted and adjusted odds of hospitalization by practitioner-assisted, self-administered, and both practitioner-assisted and self-administered CAM use compared with non-CAM use. Individual multivariable logistic models were constructed to predict each of 15 diagnostic ICD-9-CM categories for both inpatient and outpatient visits by CAM use. All models were adjusted for the following covariates: sex, birth year, race, education, marital status, military pay grade, service branch, military occupation, BMI, smoking status, and alcohol-related problems. Regression diagnostics using a variance inflation factor of four or greater were used to assess multicollinearity among the covariates [25].

Lastly, the mean number of days hospitalized as an inpatient or receiving outpatient services was compared between CAM users and nonusers. Outpatient visits were counted as one half-day for each visit. Inpatient care was counted as total number of days hospitalized that occurred anytime during the 12-month observation period. Propensity scores were calculated using logistic regression to account for baseline differences in comorbidities between CAM users and nonusers (reported in a previous study [15]). These scores were calculated and included in multivariable logistic regression to control for differences in the number of self-reported health conditions and symptoms between CAM and non-CAM users when comparing hospitalization rates [26,27]. Data management and statistical analyses were performed using SAS software, version 9.2 (SAS Institute, Inc., Cary, NC).

## Results

Of the 44,287 active-duty cohort members in this study, 29% (*n *= 12,717) reported using at least one practitioner-assisted CAM therapy, 27% (*n *= 11,996) reported using at least one self-administered CAM therapy and 61% (*n *= 26,982) reported not using any CAM therapy within the last 12 months. The frequency of the 12 CAM therapies reported for both men and women were massage therapy (24.7%), relaxation therapy (21.1%), spiritual healing (9.1%), chiropractic care (8.1%), herbal therapy (7.1%), high-dose megavitamin therapy (3.2%), folk remedies (2.3%), energy healing (1.4%), acupuncture (1.3%), homeopathic remedies, (1.3%), biofeedback (0.7%), and hypnosis (0.5%). Women reported the use of spiritual healing (13.5%) and herbal therapy (11.2%) at about twice the rate of men (7.3% and 5.5%, respectively). The other 10 CAM therapies showed similar use between men and women (data not shown).

Demographic and military characteristics of the study population by CAM use are shown in Table 1. Women reported a higher proportion of both practitioner-assisted (38.4%) and self-administered CAM use (35.4%) compared with men (24.8% and 23.7%, respectively). Reporting practitioner-assisted CAM therapies was highest among the following: women, younger individuals, those with a high school diploma or less, those who never married or were divorced, those serving in the Marine Corps, health care workers, healthy-weight individuals, current smokers, those reporting alcohol-related problems, and those reporting having one or more health conditions or symptoms. Individuals reporting one or more health conditions were 15% more likely to report CAM use and 19% more likely to report CAM use if reporting one or more health symptoms compared to non-CAM users (see Additional file 1 for list of health symptoms and conditions). Results for self-administered CAM use were very similar to practitioner-assisted CAM, with only a few exceptions. Those reporting self-administered CAM use showed a higher percentage of use among enlisted, Navy and Coast Guard, and under-weight individuals. Both the Mental and Physical Component Summary scores derived from the SF-36V were slightly lower in CAM users compared to nonusers.

**Table 1:** Demographic and military characteristics of 2004-2006 active-duty Millennium Cohort participants by complementary and alternative medicine use (*N *= 44287)

	No CAM use *n *= 26982	Practitioner-assisted CAM^* ^**use *n *= 12717**	Self-administered CAM^*† ^**use *n *= 11996**
Characteristic	*n *(%)	*n *(%)	*n *(%)
Sex		^‡^	^‡^
Male	20533 (65.3)	7786 (24.8)	7452 (23.7)
Female	6449 (50.2)	4931 (38.4)	4544 (35.4)
Birth year		^‡^	^‡^
Pre-1960	2587 (63.9)	970 (23.9)	1011 (25.0)
1960-1969	7915 (64.2)	3114 (25.2)	2943 (23.9)
1970-1979	7823 (60.5)	3790 (29.3)	3526 (27.3)
1980 and later	8657 (57.8)	4843 (32.3)	4516 (30.2)
Race/ethnicity			
White, non-Hispanic	18065 (60.8)	8517 (28.7)	8100 (27.3)
Black, Non-Hispanic	3735 (60.7)	1787 (29.1)	1668 (27.1)
Other	5177 (61.5)	2412 (28.7)	2227 (26.5)
Missing data	5 (0.0)	1 (0.0)	1 (0.0)
Education level		^‡^	^‡^
High school diploma or less	17841 (60.4)	8685 (29.4)	8317 (28.2)
Some college	3769 (61.6)	1633 (26.7)	1605 (26.2)
Bachelor's degree	2871 (61.9)	1311 (28.3)	1155 (24.9)
Graduate school	2498 (62.7)	1086 (27.3)	918 (23.1)
Missing data	3 (0.0)	2 (0.0)	1 (0.0)
Marital status		^‡^	^‡^
Never married	9693 (57.0)	5542 (32.6)	5215 (30.7)
Married	16076 (64.1)	6451 (25.7)	6095 (24.3)
Divorced	1213 (55.2)	724 (32.9)	686 (31.2)
Military pay grade			^‡^
Officer	3957 (63.1)	1743 (27.8)	1386 (22.1)
Enlisted	23025 (60.6)	10974 (28.9)	10610 (27.9)
Service branch		^‡^	^‡^
Army	10368 (59.8)	5056 (29.2)	4910 (28.3)
Navy and Coast Guard	5877 (59.1)	2987 (30.1)	2910 (29.3)
Marine Corps	1733 (59.9)	918 (31.7)	745 (25.7)
Air Force	9004 (63.7)	3756 (26.6)	3431 (24.3)
Military occupation		^‡^	^‡^
Combat specialists	4955 (62.5)	2186 (27.6)	1992 (25.1)
Electronic equip. repair	2802 (64.8)	1120 (25.9)	1057 (24.4)
Comm/intelligence	2531 (58.8)	1346 (31.3)	1226 (28.5)
Health care	2326 (52.0)	1573 (35.2)	1573 (35.2)
Other technical/allied	824 (59.5)	405 (29.3)	392 (28.3)
Functional support/admin	4915 (60.0)	2380 (29.1)	2266 (27.7)
Elec/mech equip. repair	4685 (64.8)	1829 (25.3)	1765 (24.4)
Craft workers	766 (62.8)	340 (27.9)	303 (24.9)
Service and supply	2490 (60.9)	1194 (29.2)	1123 (27.5)
Students, trainees/other	687 (59.7)	344 (29.9)	299 (26.0)
Body mass index		^‡^	^‡^
Underweight	191 (57.7)	96 (29.0)	106 (32.0)
Healthy weight	9298 (58.5)	4922 (31.0)	4616 (29.0)
Overweight	12417 (62.0)	5531 (27.6)	5236 (26.2)
Obese	3298 (61.4)	1502 (27.9)	1454 (27.1)
Missing data	1778 (6.6)	666 (5.2)	584 (4.9)
Smoking status		^‡^	^‡^
Nonsmoker	14286 (61.5)	6570 (28.3)	6099 (26.2)
Past smoker	3465 (61.6)	1504 (26.7)	1540 (27.4)
Current smoker	8071 (58.8)	4238 (30.9)	3983(29.0)
Missing data	1160 (4.3)	405 (3.2)	374 (3.1)
Alcohol-related problems^§^		^‡^	^‡^
No	24567 (61.7)	10490 (26.3)	10529 (26.4)
Yes	2415 (54.1)	1472 (33.0)	1467 (32.9)
Health conditions^||^		^‡^	^‡^
No	16784 (60.9)	6332 (23.0)	5810 (21.1)
Yes	10198 (60.9)	6385 (38.1)	6186 (37.0)
Health symptoms^||^		^‡^	^‡^
No	12345 (60.9)	3696 (18.2)	3517 (17.3)
Yes	14637 (61.0)	9021 (37.6)	8479 (35.3)

	Mean (SD)	Mean (SD)	Mean (SD)

Mental Component Summary ^¶^	51.7 (9.4)	50.3 (10.2)	49.7 (10.6)
Physical Component Summary ^¶^	53.8 (7.2)	51.1 (8.8)	51.6 (8.7)

*Practitioner-assisted complementary and alternative medicine (CAM) therapies include acupuncture, biofeedback, chiropractic care, energy healing, folk remedies, hypnosis, and massage.

*^†^Self-administered CAM therapies include herbal therapy, high-dose megavitamin therapy, homeopathy, relaxation, and spiritual healing.

^†^Practitioner-assisted and self-administered CAM therapy categories are not mutually exclusive.

^‡^*p *< 0.05.

^§^Alcohol-related problems were defined by endorsement of any of the following that occurred more than once during the past year: (a) you drank alcohol even after a doctor suggested stopping due to health problems; (b) you drank alcohol, were high from alcohol, or were hung over while you were working, going to school, or taking care of children or other responsibilities; (c) you missed or were late for work, school, or other activities because you were drinking or hung over; (d) you had a problem getting along with people while you were drinking; and (e) you drove a car after having several drinks or after drinking too much.

^||^No none reported, Yes one or more reported.

^¶^Mental and Physical Component Summary scores have been transformed using norm-based algorithms (mean = 50, standard deviation = 10).

A total of 1,449 first hospitalizations occurred among this active-duty cohort within 12 months of completing the Millennium Cohort questionnaire. First hospitalization rates and adjusted odds ratios for demographic and military characteristics are displayed in Table 2. Only two characteristics were statistically associated with a hospitalization independent of CAM use: being female 1.93 (95% CI: 1.69-2.19) or being a current smoker 1.20 (95% CI: 1.05-1.36). Birth year, education level and service branch were statistically significant for a lower probability of having a hospitalization during the study period when compared with their respective reference groups.

**Table 2:** First hospitalization rates and adjusted odds ratios for active-duty military personnel over a 1-year period enrolled in the Millennium Cohort Study 2004-2006 (*N *= 42896)*

	Hospitalization rate per 1,000	Adjusted	95% CI^†^
Characteristic	(*n *= 1449)	OR^†^	
Sex			
Male	28.3 (890)	1.00	
Female	48.8 (559)	1.93	1.69-2.19
Birth year			
Pre-1960	44.2 (179)	1.00	
1960-1969	37.2 (457)	0.73	0.60-0.90
1970-1979	29.9 (374)	0.47	0.38-0.59
1980 and later	31.1 (439)	0.46	0.36-0.60
Race/ethnicity			
White, non-Hispanic	33.2 (955)	1.00	
Black, Non-Hispanic	34.9 (208)	1.06	0.90-1.24
Other	35.2 (286)	1.10	0.95-1.26
Education level			
High school diploma or less	34.9 (989)	1.00	
Some college	35.0 (212)	0.75	0.62-0.89
Bachelor's degree	27.6 (126)	0.65	0.51-0.82
Graduate school	30.9 (122)	0.62	0.44-0.85
Marital status			
Never married	31.6 (514)	1.00	
Married	34.3 (839)	1.03	0.89-1.19
Divorced, widowed, separated	44.3 (96)	1.00	0.7-1.29
Military pay grade			
Enlisted	34.6 (1,270)	1.00	
Officer	28.8 (179)	1.08	0.82-1.43
Service branch			
Army	41.4 (697)	1.00	
Navy and Coast Guard	31.6 (302)	0.69	0.59-0.80
Marine Corps	18.6 (53)	0.49	0.35-0.66
Air Force	29.2 (402)	0.68	0.59-0.78
Military occupation			
Combat specialists	32.1 (251)	1.00	
Electronic equipment repair	28.8 (122)	0.89	0.70-1.12
Communications/intelligence	33.2 (138)	0.90	0.72-1.13
Health care	45.2 (190)	1.09	0.88-1.35
Other technical and allied	33.8 (45)	0.99	0.70-1.37
Functional support and admin	34.6 (272)	0.84	0.69-1.02
Electrical/mechanical equip. repair	33.0 (234)	1.02	0.83-1.24
Craft workers	18.3 (22)	0.54	0.32-0.84
Service and supply	38.2 (148)	1.01	0.81-1.26
Students, trainees, and other	24.2 (27)	0.91	0.59-1.36
Body mass index (BMI)			
Underweight	56.5 (17)	1.00	
Healthy weight	31.8 (483)	0.70	0.42-1.26
Overweight	32.8 (641)	0.78	0.47-1.42
Obese	42.7 (224)	1.00	0.59-1.83
Missing data	(84)		
Smoking status			
Nonsmoker	31.3 (704)	1.00	
Past smoker	36.5 (201)	1.09	0.92-1.29
Current smoker	36.7 (488)	1.20	1.05-1.36
Missing data	(56)		
Alcohol-related problems^‡^			
No	33.8 (1,302)	1.00	
Yes	33.7 (147)	1.02	0.84-1.24

*Excludes hospitalizations for complications of pregnancy, childbirth, and the puerperium (ICD-9-CM 630-676).

^†^ORs and associated confidence intervals from multiple logistic regression were adjusted for sex, age, education, marital status, race/ethnicity, pay grade, branch of service, occupation, BMI, smoking status, and problem drinking. CIs that exclude 1.00 were significant at the *p *< 0.05 level.

^‡^Alcohol-related problems were defined by endorsement of any of the following that occurred more than once during the past year: (a) you drank alcohol even after a doctor suggested stopping due to health problems; (b) you drank alcohol, were high from alcohol, or were hung over while you were working, going to school, or taking care of children or other responsibilities; (c) you missed or were late for work, school, or other activities because you were drinking or hung over; (d) you had a problem getting along with people while you were drinking; (e) you drove a car after having several drinks or after drinking too much. No = none reported, Yes = one or more reported.

The unadjusted first hospitalization rate for non-CAM users was 30.5 per 1,000 and 39.0 per 1,000 for CAM users. When considering self-administered and practitioner-assisted types of CAM alone and in combination, the rate for practitioner-assisted only was 38.4 per 1,000, self-administered only was 32.9 per 1,000, and both was 43.3 per 1,000 (Table 3). Unadjusted and adjusted odds ratios for first hospitalizations are also shown in Table 3. Unadjusted odds of hospitalization among CAM users was 1.29 (95% CI: 1.16-1.43). After adjusting for covariates and differences in comorbidities (propensity scores) the adjusted odds of hospitalization for CAM users compared to nonusers diminished in magnitude and became statistically nonsignificant 1.04 (95% CI: 0.93-1.17). We observed a higher probability of being hospitalized among those who reported using energy healing, chiropractic, relaxation, or massage therapies. Those using energy healing were hospitalized primarily for mental disorders, and those using chiropractic, relaxation, or massage therapy were primarily seen for diseases of the musculoskeletal system (data not shown).

**Table 3:** First hospitalization rates by CAM use, unadjusted and adjusted odds ratios for active-duty military personnel

	Hospitalization Rate per 1,000	Unadjusted		Adjusted	
CAM use	(*n *= 1,449)	OR	95% CI	**OR**^**†**^	**95% CI**^**†**^
Non-CAM Use (*n *= 26,260)	30.5 (800)	1.00		1.00	
CAM Use (*n *= 16,636)	39.0 (649)	1.29	1.16-1.43	1.04	0.93-1.17
Provider-admin only (*n *= 5,103)	38.4 (196)	1.27	1.08-1.49	1.06	0.89-1.26
Self-admin only (*n *= 4,439)	32.9 (146)	1.08	0.90-1.29	0.89	0.73-1.07
Both (*n *= 7,094)	43.3 (307)	1.44	1.26-1.64	1.13	0.97-1.30

A 1-year follow-up period from the time of enrollment into the Millennium Cohort Study 2004-2006 (*N *= 42896)*

*Excludes hospitalizations for complications of pregnancy, childbirth, and the puerperium (ICD-9-CM 630-676).

^†^ORs and associated confidence intervals from multiple logistic regression were adjusted for sex, age, education, marital status, race/ethnicity, pay grade, branch of service, occupation, body mass index, smoking status, problem drinking, and differences in comorbidity using propensity scores. CIs that exclude 1.00 were significant at the *p *< 0.05 level.

Active-duty Millennium Cohort participants were also evaluated for amount of time spent utilizing inpatient or outpatient care by CAM use. The mean number of days spent in outpatient health care for CAM users was 7.0 days and 5.9 days for non-CAM users (*p *< 0.001), while the mean number of days spent in inpatient care was 3.2 days and 3.1 days, respectively (*p *= 0.85). Thirty-nine percent of persons reporting CAM use accounted for 45.1% of outpatient care and 44.8% of inpatient care.

**Figure 1:**
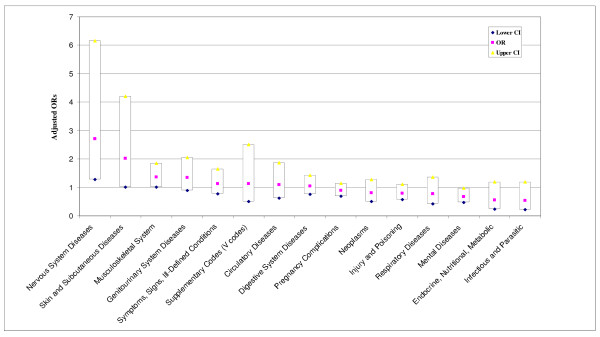
**Adjusted odds ratios and 95% confidence intervals for odds of a hospitalization visit for illnesses by CAM use versus non-CAM use, adjusted for sex, age, education, marital status, race/ethnicity, pay grade, branch of service, and occupation**.

Separate multivariable logistic regression analyses across 15 broad ICD-9-CM categories, including pregnancy and childbirth, were conducted modeling inpatient or outpatient visits by CAM use. Figure 1 illustrates each of the odds ratios for inpatient hospitalization discharge diagnoses models (excluding diseases of the blood due to sparse cases). Only hospitalization for nervous system diseases was statistically higher among those reporting CAM use compared to non-CAM users 2.72 (95% CI: 1.28-6.70). We found the majority of ICD-9-CM codes for nervous systems hospitalizations (*n *= 31) were for unspecified causes of encephalitis (ICD-9-CM 323.9), migraine unspecified (ICD-9-CM 346.9), and optic neuritis (ICD-9-CM 377.3). Mental disorders showed a slightly reduced odds ratio for hospitalization in CAM users 0.68 (95% CI: 0.47-0.97).

**Figure 2:**
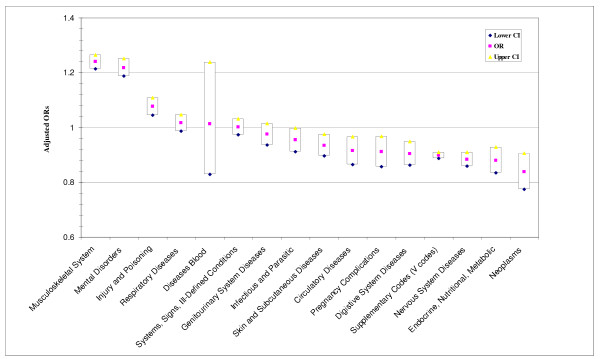
**Adjusted odds ratios and 95% confidence intervals for odds of an outpatient visit for illnesses by CAM use versus non-CAM use, adjusted for sex, age, education, marital status, race/ethnicity, pay grade, branch of service, and occupation**.

When outpatient visits were examined (Figure 2), CAM users were more likely to have had an outpatient visit for musculoskeletal system diseases 1.24 (95% CI: 1.21-1.26), mental disorders 1.22 (95% CI: 1.19-1.25), and injury and poisoning 1.08 (95% CI: 1.04-1.11). CAM users were less likely to been seen for the following: skin and subcutaneous diseases, circulatory diseases, pregnancy complications, digestive system diseases, nervous system diseases, neoplasms, and endocrine, nutritional, and metabolic disorders.

## Discussion

Our study found that those who report CAM use were disproportionally over represented in both inpatient and outpatient medical encounters compared with non-CAM users. This appears to be the result of an increase in the number of health conditions and symptoms reported by CAM users compared with non-users. In general, individuals who reported CAM use also had slightly lower Mental and Physical Component Summary scores from the SF-36V than non-CAM users, which may indicate diminished function due to poorer health [28]. Hospitalization rates were higher among CAM users for all groups except those born before 1960. As a group, these individuals appear less healthy or may perceive themselves as less healthy than their non-CAM counterparts. More importantly, CAM users appear to have greater health care requirements and tend to use both conventional and unconventional health care services. These findings are consistent with a number of other studies that have noted that CAM users tend to have more comorbid, non-life-threatening health problems than nonusers [10,11,13,14].

Previous studies have characterized typical CAM users as female, middle aged and with more education [10,29]. Although not statistically significant, we saw the opposite trend in education and age in our study, with lower levels of education and a younger age group reporting more CAM use. Our findings of higher proportions for problems of the nervous system and sense organs that required a hospitalization among CAM users is also consistent with other studies [16,17]. However, we were not able to determine if these individuals were using CAM therapies specifically for these problems. For outpatient visits, higher rates for musculoskeletal diseases, mental disorders, and injury and poisonings were seen among CAM users compared with nonusers, and is consistent with findings previously published [17,30]. However, in this study, CAM users were also less likely to been seen for skin and subcutaneous diseases, circulatory diseases, pregnancy complications, digestive system diseases, nervous system diseases, neoplasms, and endocrine, nutritional, and metabolic disorders.

Current research indicates that CAM therapies are primarily being selected by individuals to augment but not replace conventional or mainstream medicine [1,31]. Studies of CAM use in military populations consistently indicate that approximately 40% report using some form of CAM therapy [15,32,33]. Unfortunately, studies have also shown that only 35% of persons who use CAM therapies share this knowledge with their primary health care provider [5]. This is particularly important for those who take herbal therapies or nutritional supplements because of the potential for adverse drug interactions given the important pharmacological activity of some herbal therapies and nutritional supplements [34,35]. There is well-documented evidence for herbal-drug interactions in the literature, and military health care provider awareness of CAM therapies by patients may help to avoid some potential adverse reactions [36-39].

This study has limitations that should be considered when interpreting findings. First, health outcomes that occur within a 12- month study period may not represent future health care utilization patterns in this population, given their relatively young age. Because this was an active-duty population, one might expect them to have a higher level of physical fitness and have lower disease burdens than non-active-duty personnel. In addition, we did not perform a comprehensive assessment of all CAM therapies and did not capture information on the frequency or total dose of CAM therapies. Lastly, we could not assess the health care utilization patterns among Reserve or National Guard personnel since they are only eligible for DoD health care when they are on active status.

Despite these limitations there are a number of strengths with this study. Having relatively complete inpatient and outpatient records provides objective data to assess the health care utilization of our active-duty population. The results of this study were based on a sufficient sample size from one of the largest studies of active-duty personnel, CAM use, and health care utilization. To our knowledge this is the first study looking at a large military cohort in this context. Finally, because military health care is equally accessible to all active-duty service members, all study subjects have equal access to health care during the observation period, minimizing any bias associated with differential access to health care resources.

## Conclusions

Our findings provide evidence that CAM users are requiring more physician-based medical services than users of conventional care. CAM patients report a higher number of health conditions and symptoms than nonusers and have slightly lower Mental and Physical Component scores than non-CAM users. Whether CAM use represents the inability of current conventional medical practice to meet the health care needs of these individuals is not fully understood. Additional studies that include the circumstances and rationale that underlie the reasons these patients embrace CAM therapies may help to enhance conventional medical approaches.

## List of Abbreviations

BMI: Body mass index; CAM: Complementary and Alternative Medicine; CI: Confidence Interval; DoD: Department of Defense; ICD-9-CM: *International Classification of Diseases, 9th Revision, Clinical Modification*; OR: Odds Ratio; SIDR: Standard Inpatient Data Record; SF-36V: Medical Outcomes Study Short Form 36-Item Health Survey for Veterans.

## Competing interests

The authors declare that they have no competing interests.

## Authors' contributions

All authors contributed to study concept and design. MW conducted the literature review, performed the analyses and prepared major portions of the draft manuscript, and edited the final version of the manuscript. IJ, BS and TS were instrumental in obtaining the data and helping with the analysis and providing critical review of the manuscript, in addition IJ wrote and provided the SAS code for doing some of the analysis. GG, EH, TW drafted sections of the manuscript and contributed to the interpretation of the results and provided critical review of the final manuscript. All authors interpreted the data, revised the article critically for important intellectual content and approved the final version.

## Pre-publication history

The pre-publication history for this paper can be accessed here:

http://www.biomedcentral.com/1472-6882/11/27/prepub

## Supplementary Material

Additional file 1 **Appendix**. List of self-reported health conditions and symptoms assessed on the Millennium Cohort questionnaire Click here for file
