# Supplementary material for: Health care utilization among complementary and alternative medicine users in a large military cohort
Source: BMC Complement Altern Med. 2011 Apr 11;11:27. doi: 10.1186/1472-6882-11-27 (PMC3083384; doi:10.1186/1472-6882-11-27)
Supplement: Additional file 1 — Appendix. List of self-reported health conditions and symptoms assessed on the Millennium Cohort questionnaire [file 1472-6882-11-27-S1.DOC]

**Appendix**. List of self-reported health conditions and symptoms assessed on the Millennium Cohort questionnaire

| Health conditions |  | Health symptoms |
| --- | --- | --- |
|  |  |  |
| Sinusitis |  | Trouble sleeping |
| Hypertension |  | Severe headache |
| Migraine headaches |  | Forgetfulness |
| Depression |  | Pain in arms legs, or joints |
| Significant hearing loss |  | Sleepy all the time |
| Bladder infection |  | Unusual fatigue |
| Asthma |  | Menstrual cramps |
| Other heart conditions |  | Back pain |
| Anemia |  | Cough |
| Sleep apnea |  | Diarrhea |
| Posttraumatic stress disorder |  | Sore throat |
| Chronic bronchitis |  | Unusual muscle pain |
| Angina (chest pain) |  | Night sweats |
| Thyroid condition other than cancer |  | Shortness of breath |
| Stomach, duodenal, peptic ulcer |  | Rash or skin ulcer |
| Rheumatoid arthritis |  | Headaches |
| Neuropathy-caused reduced sensation |  | Chest pain |
| Cancer |  | Nausea, gas, or indigestion |
| Gallstones |  | Confusion |
| Diabetes or sugar diabetes |  | Constipation, loose bowels, or diarrhea |
| Chronic fatigue syndrome |  | Fever |
| Manic-depressive disorder |  | Sudden unexplained hair loss |
| Coronary heart disease |  | Stomach pain |
| Seizures |  | Frequent bladder infections |
| Ulcerative colitis or proctitis |  | Pain during sexual intercourse |
| Any other hepatitis |  | Feeling your heart pound or race |
| Heart attack |  | Earlobe pain |
| Hepatitis B |  | Dizziness |
| Hepatitis C |  | Fainting spells |
| Pancreatitis |  |  |
| Emphysema |  |  |
| Stroke |  |  |
| Schizophrenia or psychosis |  |  |
| Multiple sclerosis |  |  |
| Lupus |  |  |
| Crohn’s disease |  |  |
| Kidney failure requiring dialysis |  |  |
